# The dialysis facility levels and sizes are associated with outcomes of incident hemodialysis patients

**DOI:** 10.1038/s41598-021-00177-x

**Published:** 2021-10-18

**Authors:** George Kuo, Tao-Han Lee, Jia-Jin Chen, Chieh-Li Yen, Pei-Chun Fan, Cheng-Chia Lee, Chih-Hsiang Chang

**Affiliations:** 1 Department of Nephrology, Kidney Research Center, Linkou Chang Gung Memorial Hospital, No.5, Fuxing Street, Guishan District, Taoyuan, 33305 Taiwan; 2 College of Medicine, Chang Gung University, Taoyuan, Taiwan

**Keywords:** Renal replacement therapy, Epidemiology, Risk factors

## Abstract

The outcomes of patients with incident kidney failure who start hemodialysis are influenced by several factors. Whether hemodialysis facility characteristics are associated with patient outcomes is unclear. We included adults diagnosed as having kidney failure requiring hemodialysis during January 1, 2001 to December 31, 2013 from the Taiwan National Health Insurance Research Database to perform this retrospective cohort study. The exposures included different sizes and levels of hemodialysis facilities. The outcomes were all-cause mortality, cardiovascular death, infection-related death, hospitalization, and kidney transplantation. During 2001–2013, we identified 74,406 patients and divided them in to three groups according to the facilities where they receive hemodialysis: medical center (n = 8263), non-center hospital (n = 40,008), and clinic (n = 26,135). The multivariable Cox model demonstrated that a larger facility size was associated with a low mortality risk (hazard ratio [HR] 0.991, 95% confidence interval [95% CI] 0.984–0.998; every 20 beds per facility). Compared with medical centers, patients in non-center hospitals and clinics had higher mortality risks (HR 1.13, 95% CI 1.09–1.17 and HR 1.11, 95% CI 1.06–1.15, respectively). Patients in medical centers and non-center hospitals had higher risk of hospitalization (subdistribution HR [SHR] 1.11, 95% CI 1.10–1.12 and SHR 1.22, 95% CI 1.21–1.23, respectively). Patients in medical centers had the highest rate of kidney transplantation among the three groups. In patients with incident kidney failure, a larger hemodialysis facility size was associated with lower mortality. Overall, medical center patients had a lower mortality rate and higher transplantation rate, whereas clinic patients had a lower hospitalization risk.

## Introduction

Patients with kidney failure who require dialysis usually have multiple comorbidities and are at a risk of cardiovascular events and infection. The 5-year survival of patients with kidney failure is approximately 40–60% in different health care systems and countries^1,2^; the highest rate of mortality usually occurs within the first 12 months following dialysis initiation^3^. The short- to medium-term outcomes of patients with kidney failure after dialysis initiation may be influenced by many patient factors including their age, sex, comorbidities, and the hemodialysis access types. The facility factors may also play roles in the outcomes of hemodialysis patients. The facility factors include facility size, facility level, health insurance program, and health care accessibility^4–9^.

The associations between facility factors and patient outcomes have been reported in patients requiring procedures or surgeries, such as coronary intervention, cardiac, major abdominal and plastic surgery^10,11^. This association could be attributed to experienced operators, trained staff, well-maintained equipment, well-established processes, and facilities^4,12–14^. The association between facility size and outcome in hemodialysis was reported in the Unites States, but the level of facility was not analyzed^4^.

The implementation of Taiwan’s National Health Insurance (NHI), a mandatory health insurance system established in 1995, made health care services highly accessible and affordable. Because of the advantage of NHI and the low rate of organ donation and transplantation, approximately 90,000 patients with prevalent kidney failure undergo dialysis in Taiwan. Between both dialysis modalities, patients who choose peritoneal dialysis (PD) are usually followed up at the hospital level, whereas hemodialysis (HD) can be easily performed in both hospitals and clinics. The levels and sizes of HD facilities vary widely; nevertheless, they are all reimbursed by the NHI.

In this study, we analyzed patient and facility factors that may have affected the short- and mid-term outcomes of patients with incident kidney failure who initiate HD therapy.

## Materials and methods

### Data source and ethic declarations

This study was performed using data from the Taiwan National Health Insurance Research Database (NHIRD), which has > 99.8% coverage of Taiwan’s 23 million residents. This database contains deidentified data collected since 1996 and includes information on inpatient and outpatient services, diagnoses, examinations, prescriptions, and operations. The comorbidities, modality details, and outcomes of patients were obtained by using *International Classification of Diseases, Ninth Revision, Clinical Modification* (ICD-9-CM) codes and Taiwan’s NHI procedure codes from inpatient and outpatient claims data. Detailed information on database has been described in previous studies^15–17^. The study was approved by the Chang Gung Medical Foundation Institutional Review Board and was performed in accordance with the it’s regulation and the Declaration of Helsinki. The need for informed consent was waived by the Chang Gung Medical Foundation Institutional Review Board because of the study’s retrospective design and the use of deidentified data.

### Study cohort

**Figure 1:**
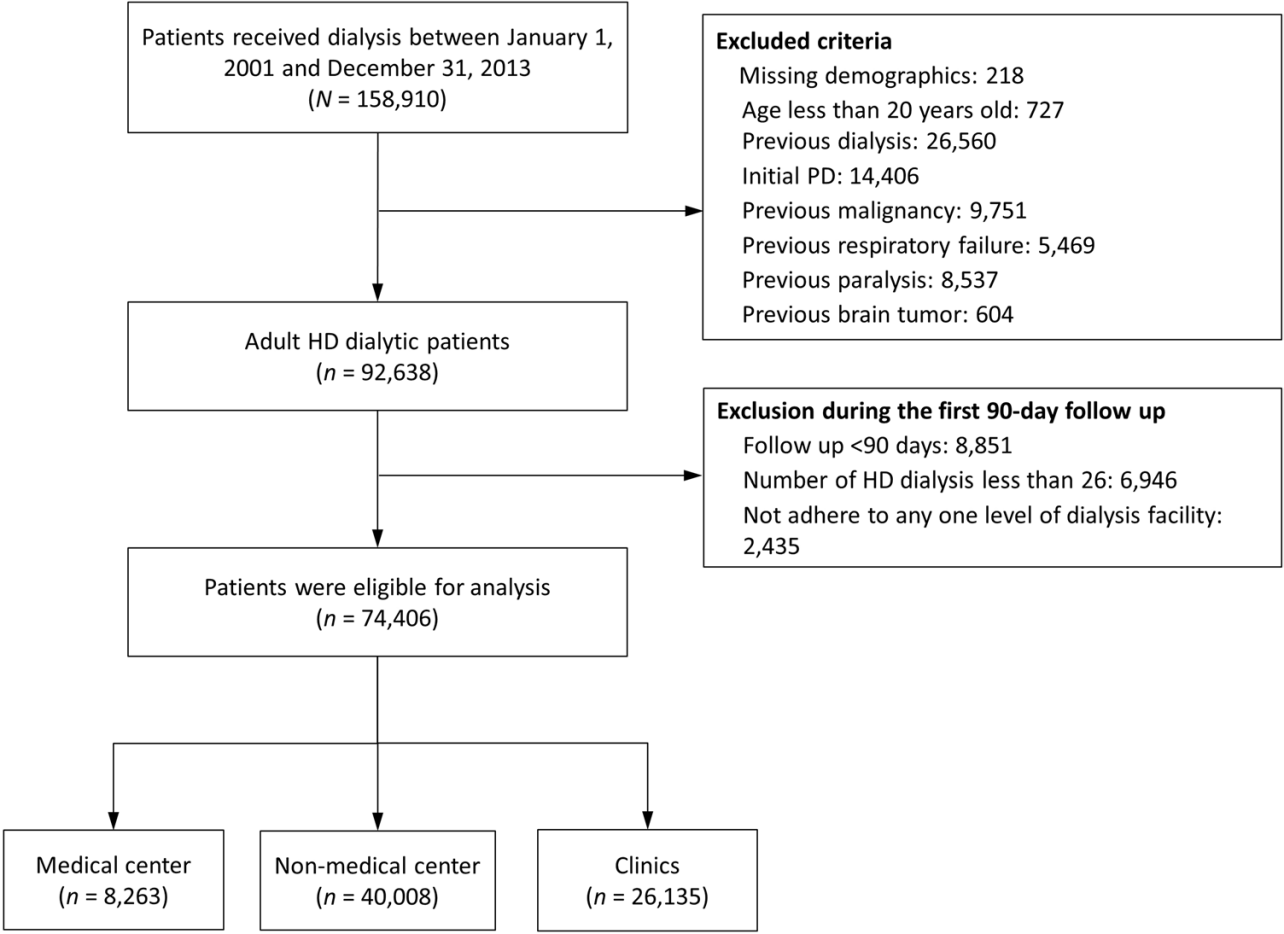
Flowchart for patient selection.

We identified patients with incident kidney failure with replacement therapy (KFRT) who started HD during January 1, 2001 to December 31, 2013. The kidney failure diagnosis was verified based on possession of a catastrophic illness certificate (CIC)^18^. The application date of CIC was defined as the index date. We excluded patients aged < 20 years old, those who chose PD as the initial modality, those with malignancy, those who received any dialysis therapy before the index date, and those who had severe diseases that may have led to a long-term bed confinement status or ventilator dependency. Patients with a follow-up duration < 90 days (including death during this period), who did not stably receive HD, and who did not receive two third of their HD sessions at any one level of dialysis facility were excluded (Fig. 1).

### Covariates

We selected covariates of demographics (age, sex, residence urbanization level, and monthly income range), health resource utilization (number of nephrologist outpatient visits in the past year), facility factors (facility level and size), and initial vascular access. Comorbidities were identified based on the presence of corresponding ICD-9-CM codes twice in outpatient records or once in inpatient records within 1 year before the index date. The presence of an event history was determined using inpatient diagnoses before the index date, which can be tracked up to year 1997. Most of the diagnoses of comorbidities and events were validated in previous studies^19,20^. Initial vascular access information was captured using the Taiwan NHI reimbursement codes from outpatient and inpatient claims data.

### Facility levels

With the regulation by the Ministry of Health and Welfare in Taiwan, the criteria of medical center is based on the inpatient bed number (more than 250 beds of acute medical and surgical services, plus more than 25 beds of acute psychiatric service), the level of emergent and critical care services (e.g. the ability to provide 24-h primary coronary intervention, the ability to take care of extracorporeal membrane oxygenation, major trauma, organ transplantation, etc.), with accreditation of comprehensive cancer treatment, the accreditation as a teaching hospital, and the completeness of subspecialty services. The dialysis centers are operated by different level of healthcare facilities, and we stratified them into 3 levels, namely medical centers, non-center hospitals, and clinics, based on the Institution Accreditation subdatabase in the NHIRD. Each hemodialysis session receives same reimbursement regardless of the facility levels. The accreditation of hemodialysis service further regulates the physician–patient and staff–patient ratio. The dialysis facility size was presented as HD bed numbers, which was extracted from the Institution Bed subdatabase in the NHIRD.

### Outcomes

The outcomes were all-cause mortality, cardiovascular death, infection-related death, major cardiac and cerebrovascular events (MACCEs), all-cause admission, infection-related hospitalization, and kidney transplantation during the 2-year follow-up and at the end of the follow-up. Death was indicated by withdrawal from the NHI program^21^. The cause of death was determined using the main diagnosis in discharge records for inpatient hospital deaths, the primary diagnosis of the last emergency room visit, or the cause of hospitalization within 7 days of death for out-of-hospital deaths. MACCEs were acute myocardial infarction (AMI), ischemic stroke, and cardiovascular death. AMI and ischemic stroke were defined by the principal diagnosis on admission. The date of kidney transplantation is detected by using the NHI reimbursement codes of the inpatient claims data. Kidney transplantation status was confirmed by the presence of ICD-9-CM code V42.0 in the outpatient diagnoses with a corresponding immunosuppressant prescription after the kidney transplantation date. All patients were followed from the index date to the event occurrence date, date of withdrawal from the NHI program, the day of kidney transplantation, or December 31, 2013, whichever came first.

### Statistical analysis

This was a retrospective cohort study and the distribution of patient’s characteristics among different facility levels might be substantially different. To mitigate the possible selection bias, we applied an inverse probability of treatment weighting (IPTW) of propensity scores (PSs) when comparing the outcomes among different dialysis facility levels. To obtain optimal balance between dialysis facility levels, the PSs were estimated using the generalized boosted model (GBM), which was based on 50,000 regression trees^22^. Explanatory variables included in the PS estimation were all the covariates listed in Table 1, except that the follow-up duration was replaced with the index date. Dependent variables in the PS estimation were the facility levels (1 = medical center, 2 = non-medical center hospital, and 3 = clinics). The balance among different dialysis facility levels before and after GBM-IPTW was investigated using standardized differences^23^; a threshold of > 0.1 indicated an imbalance.

**Table 1:** Baseline characteristics of patients according to the dialysis facility level before and after IPTW.

Variable	Before IPTW^a^			After IPTW^b^			
	Medical center (*n* = 8263)	Non-medical center (*n* = 40,008)	Clinics (*n* = 26,135)	Medical center	Non-medical center	Clinics	MASD
Age (years)	60.3 ± 14.0	63.2 ± 13.6	63.4 ± 13.5	62.8 ± 13.3	63.0 ± 13.6	63.0 ± 13.5	0.01
**Age group (years)**							
20–64	5053 (61.2)	20,741 (51.8)	13,510 (51.7)	53.4	52.7	52.8	0.01
65–74	1893 (22.9)	10,724 (26.8)	7001 (26.8)	26.7	26.5	26.5	0.01
≥ 75	1317 (15.9)	8543 (21.4)	5624 (21.5)	19.9	20.8	20.7	0.02
Male	4394 (53.2)	20,505 (51.3)	13,574 (51.9)	51.1	51.7	51.7	0.01
No. of nephrologist outpatient visits in the previous year	11.7 ± 9.2	8.9 ± 8.9	9.8 ± 9.1	9.7 ± 8.9	9.5 ± 9.0	9.5 ± 9.0	0.02
**Urbanization level**							
Low	333 (4.0)	5814 (14.5)	3057 (11.7)	11.4	12.4	12.3	0.03
Moderate	1918 (23.2)	13,705 (34.3)	8533 (32.6)	31.5	32.5	32.5	0.02
High	2696 (32.6)	12,121 (30.3)	7384 (28.3)	31.1	29.9	29.8	0.03
Very High	3316 (40.1)	8368 (20.9)	7161 (27.4)	26.1	25.2	25.4	0.02
**Monthly income, US dollars**							
0–593	3161 (38.3)	15,064 (37.7)	10,235 (39.2)	37.8	38.2	38.3	0.01
596–760	2466 (29.8)	15,688 (39.2)	9608 (36.8)	37.0	37.4	37.4	0.01
> 760	2636 (31.9)	9256 (23.1)	6292 (24.1)	25.2	24.4	24.3	0.02
**Comorbidity**							
Diabetes mellitus	4375 (52.9)	23,553 (58.9)	15,428 (59.0)	58.5	58.3	58.5	< 0.01
Hypertension	7161 (86.7)	35,146 (87.8)	23,156 (88.6)	88.1	88.0	88.1	< 0.01
Dyslipidemia	2381 (28.8)	10,245 (25.6)	7114 (27.2)	26.7	26.4	26.4	0.01
Coronary arterial disease	2126 (25.7)	11,208 (28.0)	6978 (26.7)	27.5	27.3	27.1	0.01
Peripheral arterial disease	221 (2.7)	1377 (3.4)	880 (3.4)	3.3	3.3	3.3	< 0.01
Chronic obstructive pulmonary disease	532 (6.4)	3316 (8.3)	1901 (7.3)	7.1	7.7	7.7	0.02
Obstructive sleep apnea	31 (0.4)	113 (0.3)	73 (0.3)	0.23	0.28	0.27	0.01
Gouty arthritis	1570 (19.0)	6882 (17.2)	4446 (17.0)	17.5	17.3	17.2	0.01
Liver cirrhosis	277 (3.4)	1629 (4.1)	957 (3.7)	3.5	3.8	3.9	0.02
**History of event**							
Heart failure hospitalization	1582 (19.1)	10,732 (26.8)	6321 (24.2)	24.6	25.1	25.0	0.01
Ischemic stroke	830 (10.0)	5693 (14.2)	3446 (13.2)	12.6	13.5	13.3	0.03
Hemorrhage stroke	92 (1.1)	680 (1.7)	464 (1.8)	1.4	1.7	1.7	0.02
Systemic embolism	205 (2.5)	1205 (3.0)	838 (3.2)	2.8	3.0	3.1	0.01
Myocardial infarction	474 (5.7)	2385 (6.0)	1774 (6.8)	6.4	6.2	6.3	0.01
Charlson comorbidity index	4.2 ± 1.8	4.6 ± 1.9	4.5 ± 1.9	4.5 ± 1.9	4.5 ± 1.9	4.5 ± 1.9	0.03
**Initial vascular access**							
Arteriovenous fistula	6407 (77.5)	27,450 (68.6)	18,135 (69.4)	71.5	69.7	69.9	0.04
Arteriovenous graft	396 (4.8)	3554 (8.9)	2362 (9.0)	7.7	8.5	8.5	0.03
Tunneled cuffed catheter	1460 (17.7)	9004 (22.5)	5638 (21.6)	20.8	21.7	21.6	0.02
Follow up duration (years)	5.3 ± 3.6	4.4 ± 3.3	4.0 ± 3.1	4.6 ± 3.3	4.3 ± 3.3	4.4 ± 3.3	0.02

*IPTW* inverse probability of treatment weighting, *MASD* maximum absolute standardized difference, *AV* arteriovenous.

^a^Data were presented as frequency (percentage) or mean ± standard deviation;

^b^Data were presented as percentage or mean ± standard deviation.

The association between covariates and all-cause mortality risk was studied using the multivariable Cox proportional hazards model in the original cohort before propensity score weighting. The event rate of outcomes and survival analyses were estimated in the cohort after propensity score weighting. The risk of time to event outcomes among different dialysis facility levels was compared using Cox proportional hazard model. The facility level was the only explanatory variable in the aforementioned survival analyses. A 2-sided *P* value of < 0.05 was considered statistically significant. Statistical analyses were performed using SAS version 9.4 (SAS Institute, Cary, NC)^24^.

### Ethics approval and consent to participate

The NHIRD contains no identifiable personal information; the need for informed consent was waived by the Chang Gung Medical Foundation Institutional Review Board because of this study’s retrospective noninterventional design and because patient data confidentiality and privacy were maintained. The study was approved by the Chang Gung Medical Foundation Institutional Review Board.

## Results

### Patient selection

Between January 01, 2001 and December 31, 2013, 158,910 patients with KFRT were screened. After the exclusion of patients aged < 20 years, who received dialysis before the index date, whose initial modality was PD, and who had malignancies, ventilator dependency, or bed confinement status, 92,638 adult ambulatory HD patients were included. During the first 90 days following the index date, patients who were lost to follow-up, received HD less than twice per week, or did not receive HD predominantly at a certain facility level were also excluded. A total of 74,406 patients were eligible for analysis, and they were divided into three groups based on the facility level: medical centers, non-center hospitals, and clinics (Fig. 1).

### Patient characteristics and trends of epidemiology

The HD center sizes varies in different levels of facility (mean HD bed numbers in medical center: 88.7 ± 46.7, non-center hospital: 49.6 ± 35.7 and the clinic: 28.5 ± 11.3) (data not shown). Before propensity score weighting, patients undergoing dialysis at medical centers were younger, resided in urban areas, had higher incomes, had fewer comorbidities and prior cardiovascular events, and had higher numbers of nephrology clinic visit before HD. After propensity score weighting, all covariates in the three groups had maximum absolute standardized differences of < 0.1, indicating the groups were well-balanced (Table 1). In addition, the distribution of the facility levels was balanced across the study period after propensity score weighting (Supplementary Fig. 1).

**Figure 2:**
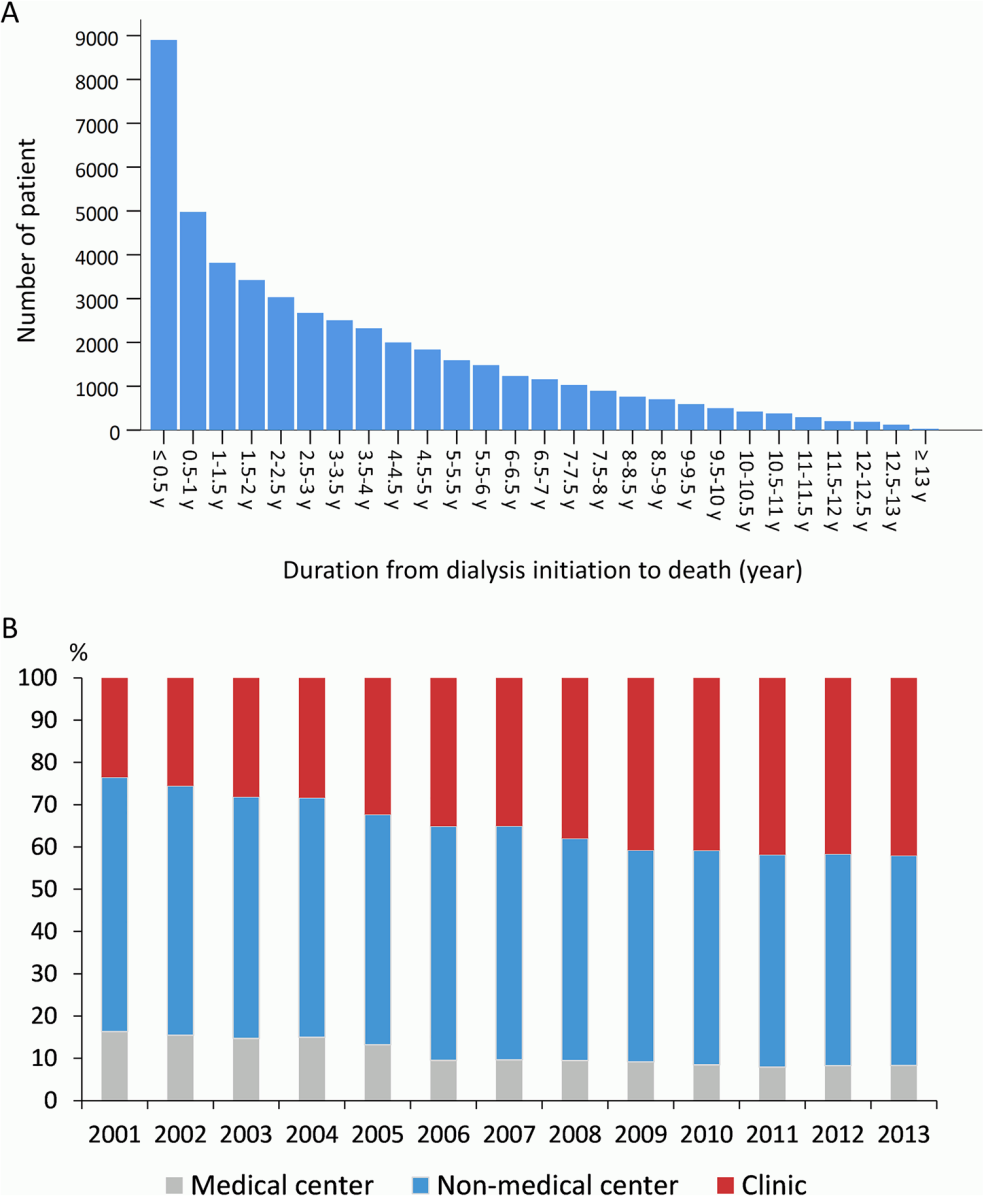
(**A**) Distribution of the duration from dialysis initiation to death and (**B**) the distribution of facility level related to initial hemodialysis during 2001–2013 in Taiwan.

Figure 2A depicts the time from HD initiation to death. Of the 92,638 PD patients, 49,176 (53.1%) died during the follow-up. Our study demonstrated that the mortality peak occurs within 1 year following HD initiation, and the mortality rate decreased steadily with time. From 2001 to 2013, the proportion of patients who received HD at clinics gradually increased (Fig. 2B).

### Risk factors of mortality

A multivariate Cox model showed that the risk factors for mortality in incident HD patients included older age, male sex, low monthly income, low urbanization area residence, diabetes mellitus, coronary arterial disease, chronic obstructive pulmonary disease, liver cirrhosis, prior cardiac and cerebrovascular events, and use of nonnative fistula access. Conversely, hypertension, dyslipidemia, hyperuricemia, gout, large HD station number, and receive HD at medical center than other levels of facility were associated with low risks of all-cause mortality (Table 2).

**Table 2:** Associated factors of all-cause mortality in the original cohort before IPTW.

Variable	HR (95% CI)	*P*
**Dialysis facility level**		
Medical center	Reference	
Non-medical center	1.13 (1.09–1.17)	< 0.001
Clinics	1.11 (1.06–1.15)	< 0.001
Dialysis facility size (per 20 beds)	0.991 (0.984–0.998)	0.009
Age (per year)	1.05 (1.05–1.05)	< 0.001
Male	1.20 (1.17–1.23)	< 0.001
No. of nephrologist outpatient visits in the previous year	0.987 (0.986–0.988)	< 0.001
**Urbanization level**		
Low	Reference	
Moderate	0.96 (0.92–0.99)	0.008
High	0.94 (0.91–0.98)	0.001
Very High	0.967 (0.931–1.004)	0.081
**Monthly income, US dollars**		
0–593	Reference	
596–760	0.95 (0.93–0.98)	< 0.001
> 760	0.87 (0.85–0.90)	< 0.001
Diabetes mellitus	1.34 (1.30–1.38)	< 0.001
Hypertension	0.95 (0.92–0.98)	0.002
Dyslipidemia	0.95 (0.92–0.97)	< 0.001
Coronary arterial disease	1.04 (1.02–1.07)	0.001
Peripheral arterial disease	1.05 (0.99–1.11)	0.110
Chronic obstructive pulmonary disease	1.09 (1.05–1.13)	< 0.001
Obstructive sleep apnea	1.03 (0.85–1.25)	0.769
Gouty arthritis	0.92 (0.89–0.94)	< 0.001
Liver cirrhosis	1.50 (1.43–1.58)	< 0.001
Heart failure hospitalization	1.20 (1.17–1.23)	< 0.001
Ischemic stroke	1.20 (1.16–1.23)	< 0.001
Hemorrhage stroke	1.20 (1.11–1.30)	< 0.001
Systemic embolism	1.29 (1.21–1.37)	< 0.001
Myocardial infarction	1.15 (1.11–1.20)	< 0.001
Charlson comorbidity index	1.09 (1.08–1.10)	< 0.001
**Initial vascular access**		
Arteriovenous fistula	Reference	
Arteriovenous graft	1.17 (1.13–1.21)	< 0.001
Tunneled cuffed catheter	1.28 (1.24–1.31)	< 0.001

*IPTW* inverse probability of treatment weighting, *AV* arteriovenous, *HR* hazard ratio, *CI* confidence interval.

### Comparison of outcomes between facility levels

**Figure 3:**
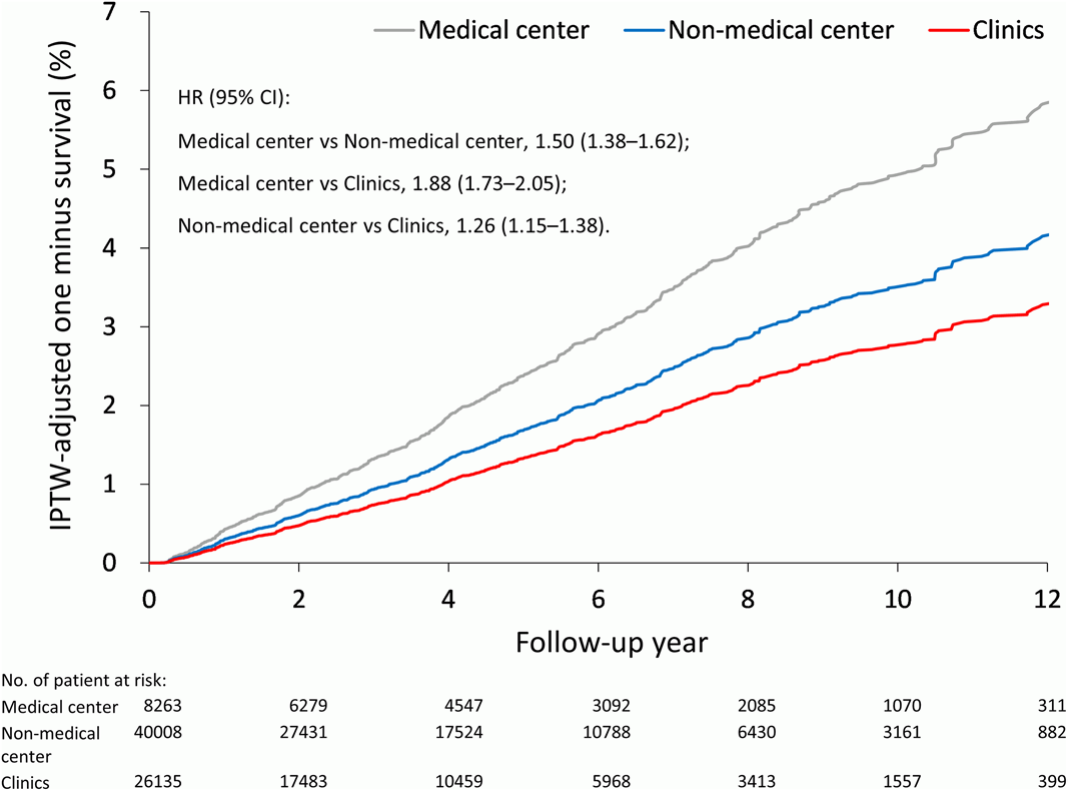
IPTW-adjusted rate of kidney transplantation for patients at different facility levels for initial hemodialysis. *IPTW* inverse probability of treatment weighting.

After propensity score weighting, the time-to-event analysis showed that patients who attended medical centers had the lowest risks of all-cause mortality, cardiovascular death, and infection-related death, whereas patients who attended clinics had the lowest risk of all-cause and infection-related hospitalization. Medical center patients were more likely to receive kidney transplantation than non-center hospital patients (hazard ratio [HR] 1.42, 95% confidence interval [CI] 1.31–1.53) and clinic patients (HR 1.80, 95% CI 1.65–1.96). Furthermore, patients who attended non-center hospitals were more likely to receive kidney transplantation than clinic patients (HR 1.27, 95% CI 1.16–1.39). The detailed outcomes in different groups were summarized in Table 3. The propensity score weighting-adjusted rate of kidney transplantation among different facility levels is illustrated in Fig. 3.

**Table 3:** Outcome of the patients according to the dialysis facility level during the follow up after IPTW.

Outcome	Number of event (%)			HR or SHR (95% CI)		
	Medical center	Non-medical center	Clinics	Medical center vs. non-medical center	Medical center vs. clinics	Non-medical center vs. clinics
**All-cause mortality**						
2 year	14.5	17.6	16.9	0.80 (0.78–0.82)*	0.84 (0.82–0.86)*	1.05 (1.02–1.07)*
Overall	45.5	48.8	48.6	0.87 (0.86–0.89)*	0.89 (0.87–0.90)*	1.015 (0.999–1.03)
**Cardiovascular death**						
2 year	7.7	8.7	8.5	0.86 (0.83–0.90)*	0.89 (0.86–0.92)*	1.03 (0.99–1.07)
Overall	23.4	23.9	23.8	0.92 (0.90–0.94)*	0.93 (0.91–0.95)*	1.02 (0.99–1.04)
**Infection-related death**						
2 year	7.0	8.3	7.5	0.82 (0.79–0.86)*	0.91 (0.88–0.95)*	1.11 (1.07–1.15)*
Overall	19.7	22.0	20.6	0.84 (0.82–0.86)*	0.91 (0.89–0.93)*	1.08 (1.06–1.10)*
**MACCE**^a^						
2 year	13.1	13.9	13.2	0.93 (0.90–0.96)*	0.98 (0.96–1.01)	1.06 (1.03–1.09)*
Overall	32.2	32.7	31.8	0.97 (0.95–0.98)*	1.00 (0.99–1.02)	1.04 (1.02–1.06)*
**All-cause admission**						
2 year	65.4	69.1	61.9	0.89 (0.88–0.91)*	1.12 (1.10–1.13)*	1.25 (1.23–1.26)*
Overall	82.9	85.0	81.2	0.90 (0.89–0.91)*	1.10 (1.09–1.11)*	1.22 (1.20–1.23)*
**Infection-related hospitalization**						
2 year	35.1	39.9	32.2	0.84 (0.83–0.85)*	1.11 (1.09–1.13)*	1.32 (1.30–1.35)*
Overall	57.3	60.6	54.0	0.88 (0.86–0.89)*	1.09 (1.08–1.11)*	1.25 (1.23–1.26)*
**Renal transplantation**						
2 year	0.72	0.53	0.41	1.32 (1.16–1.51)*	1.73 (1.50–2.00)*	1.31 (1.12–1.52)*
Overall	2.2	1.4	1.2	1.42 (1.31–1.53)*	1.80 (1.65–1.96)*	1.27 (1.16–1.39)*

*HR* hazard ratio, *SHR* subdistribution hazard ratio, *CI* confidence interval, *MACCE* major adverse cardiac and cerebrovascular events.

Data were presented as percentage.

*P value < 0.05;

^a^Including acute myocardial infarction, acute ischemic stroke and cardiovascular death.

## Discussion

Our study revealed that advanced age, low socioeconomic status, higher comorbidities, vascular types other than fistula at the initial access, non-medical center based facilities and smaller facility size were associated with worse short- and long-term outcomes of adult incident HD patients in Taiwan.

Several risk factors for mortality, including DM, CAD, COPD, liver cirrhosis, and prior cardiac events, were consistently reported in previous large registries or cohorts^3–6,13,25–27^. The overall comorbidity burden, collectively presented as the Charlson Comorbidity Index, was associated with mortality risk. By contrast, hypertension, dyslipidemia, and hyperuricemia/gout were associated with low mortality in our study. This could be explained by reverse epidemiology finding in the dialysis population in which relatively high blood pressure, plasma cholesterol, and uric acid levels usually also represent a favorable nutritional status rather than risk factors^28–30^.

The level and station number of the HD facility were associated with mortality. Large HD bed numbers usually represent a large dialysis service group with more resources in staff training, continued education, and interdisciplinary care. Taiwan’s NHI is a single-payer healthcare program with mandatory enrollment that covers > 99.8% of Taiwan’s 23 million residents. With this single-payer system, the overall reimbursement is positively correlated with service volume. However, the fixed cost per service may decrease when the service volume increases to a significant level. The reduction of fixed costs in large service groups may make them resource abundant. In 2013, Yan et al. analyzed data from the USRDS to investigate the relationship among HD facility size, ethnicity, and mortality. By using 26–30 HD beds as a reference, an HD station number below this level was associated with a high mortality risk^4^. Our study found that medical center patients had a higher survival benefit than non-center hospital and clinic patients; however, medical center and non-center hospital patients also had a higher hospitalization rate than the clinic patients. This result has not been previously reported and may be explained through several factors. Because the accessibility of health care services for patients who received HD at medical centers is high, they may receive a diagnosis of cardiovascular disease or a confirmation of infection at an early stage. HD patients were at a high risk of MACCEs wherever they receive dialysis therapy, and hence, a dialysis facility where emergency response staff are well-trained, fully available, and can provide services in a timely manner may influence patient survival. For example, Anderson et al. reported that patients with cardiac arrest who received in-hospital resuscitation were more likely to survive than those who received out-of-hospital resuscitation^31^. As for AMI, shortening door-to-balloon time to < 60 min can improve survival compared with a door-to-balloon time of 60–90 min^32^. Such a short door-to-balloon time is undoubtedly more likely to be achieved in medical center patients. The same reason also applies to infection. Liu et al., in a large retrospective cohort study involving 35,000 patients with sepsis, demonstrated that for every 1-h delay in the administration of appropriate antibiotics on sepsis recognition, the relative mortality risk increased by 9%^33^. These findings indicate that in a medical center, emergent situations could be managed faster than at other non-center facilities owing to the lack of need for transportation.

Despite the extensive control of possible confounding factors, some unmeasured factors were present. From our daily practice, we have observed that many frail patients who chose to receive HD at non-center hospitals or clinics in terms of proximity. Frail elderly patients and non-frail ambulatory patients may have similar numbers of comorbidities, but frailty is an independent risk factor for mortality^34,35^. The extent of frailty could not be adjusted in most claims databases, including Taiwan’s NHIRD, and this may contribute to more adverse events in facilities where patients’ frailty is higher. Another inadequately adjusted factor could be socioeconomic status. Almost all medical centers in Taiwan are located in highly urbanized areas, and patients who received regular HD at medical centers usually have a higher income than others. The differences in income could not be adequately controlled because the upper limit of incomes in Taiwan’s NHIRD is below the average monthly regular earnings of all employees (US$760 vs US$1400), which makes the scale of income less sophisticated^36,37^. For example, a patient living in urban area, earning 3,000 USD per month and receiving HD in the medical center will be categorized into similar income group with another patient living in countryside, earning 1000 USD per month and receiving HD in the clinic.

Despite a low mortality rate, all-cause and infection-related hospitalizations were the highest in the medical center group than in the other two groups. Approximately half of the hospitalizations in the three groups were infection related. Although all healthcare facilities require accreditation for infection control, the hospitals may be more difficult to keep clean because the complexity of patient flow compared with a simple, primary clinic. The larger bed numbers in the hospital-based dialysis makes the possibility of patient–patient or staff–patient transmission of infectious disease much higher. Meanwhile, the possibility of antibiotic resistance is higher in hospitals than in primary care settings. These factors may explain the result that patients who received HD at a hospital level had a high infection risk^38,39^. Conversely, an increased hospitalization rate with reduced mortality imply that medical center patients may receive more meticulous care with regard to their health problems. However, the balance among hospitalization, life quality, and mortality remained unanswered in our study.

The transplantation rate is significantly higher for medical center patients than in non-center hospital and clinic patients. Studies have focused on different factors related to kidney transplantation referral; however, the association between kidney transplantation rate and dialysis facility level had never been reported. In a study investigating factors associated with transplantation referral rates in the United States, Patzer et al. found that the referral rate was high in young patients with less comorbidities who received dialysis in a facility that was nonprofit or had a high social worker–patient ratio^40^. In Taiwan, most kidney transplantation centers are also medical centers. Medical center patients may receive more information about transplantation, be advised to register for transplantation, and eventually receive a kidney allograft.

This study has limitations. First, because of a lack of laboratory data and frailty scores, we were unable to adjust for these factors. However, a laboratory or severity score at single time point could not correctly predict mid- or long-term outcomes. Second, this database did not have information regarding dialysis adequacy. Survival was worse in patients whose single pool Kt/V was < 0.8, as shown in the National Cooperative Dialysis Study^41^. Nevertheless, in the HEMO study, achieving Kt/V of > 1.7 did not confer better survival than did a Kt/V of 1.3^42^. The calculation of Kt/V is subject to some bias such as under- and over-estimation in overweight and underweight patients, respectively. The use of Kt/V to guide dialysis prescription has also encountered criticisms. Despite a lack of aforementioned lab data, we use propensity score weighting to balance the patient related factors among three groups. The propensity score weighting has a greater statistical power than matching, and it also has a better control of confounding than traditional multivariable adjustment. Third, because of the complex data cleaning, validation and de-identifying process with resultant long lag time in NHIRD, we only obtain data from 2001 to 2013. However, the reimbursement and contents of hemodialysis service has not substantially changed in the last 10 years in Taiwan, we believed that our result would not be affected too much without the latest data.

In conclusion, the study demonstrated that larger HD facility sizes and medical center-based HD facilities were associated with lower all-cause mortality after adjustment for multiple patient factors. Compared with clinic patients, patient in medical center tended to have lower mortality rates but higher hospitalization rates. Patients in medical centers received transplantation more often than did clinic patients. Under the single-payer health care systems in Taiwan, the relocation of resources may help minimize the discrepancy of health-related outcomes in HD patients who are treated at facilities of different sizes or levels.

## Supplementary Information

Supplementary Legends.

Supplementary Figure S1.

## Data Availability

The data underlying this study are from the National Health Insurance Research Database (NHIRD), which have been transferred to the Health and Welfare Data Science Center (HWDC). Interested researchers can obtain the data through formal application to the HWDC, Department of Statistics, Ministry of Health and Welfare, Taiwan (http://dep.mohw.gov.tw/DOS/np-2497-113.html). The authors have no privilege over others in obtaining the data from the NHIRD.
