# Supplementary material for: The dialysis facility levels and sizes are associated with outcomes of incident hemodialysis patients
Source: Sci Rep. 2021 Oct 18;11:20560. doi: 10.1038/s41598-021-00177-x (PMC8523705; doi:10.1038/s41598-021-00177-x)
Supplement: Supplementary file 1 — Supplementary Legends. [file 41598_2021_177_MOESM1_ESM.docx]

**Supplemental Figure Legend**

**Supplemental Figure 1.** The distribution of facility level related to initial hemodialysis during 2001 to 2013 in Taiwan in the IPTW-adjusted cohort.

IPTW, inverse probability of treatment weighting.
