# Supplementary figures and images for: The dialysis facility levels and sizes are associated with outcomes of incident hemodialysis patients
Source: Sci Rep. 2021 Oct 18;11:20560. doi: 10.1038/s41598-021-00177-x (PMC8523705; doi:10.1038/s41598-021-00177-x)

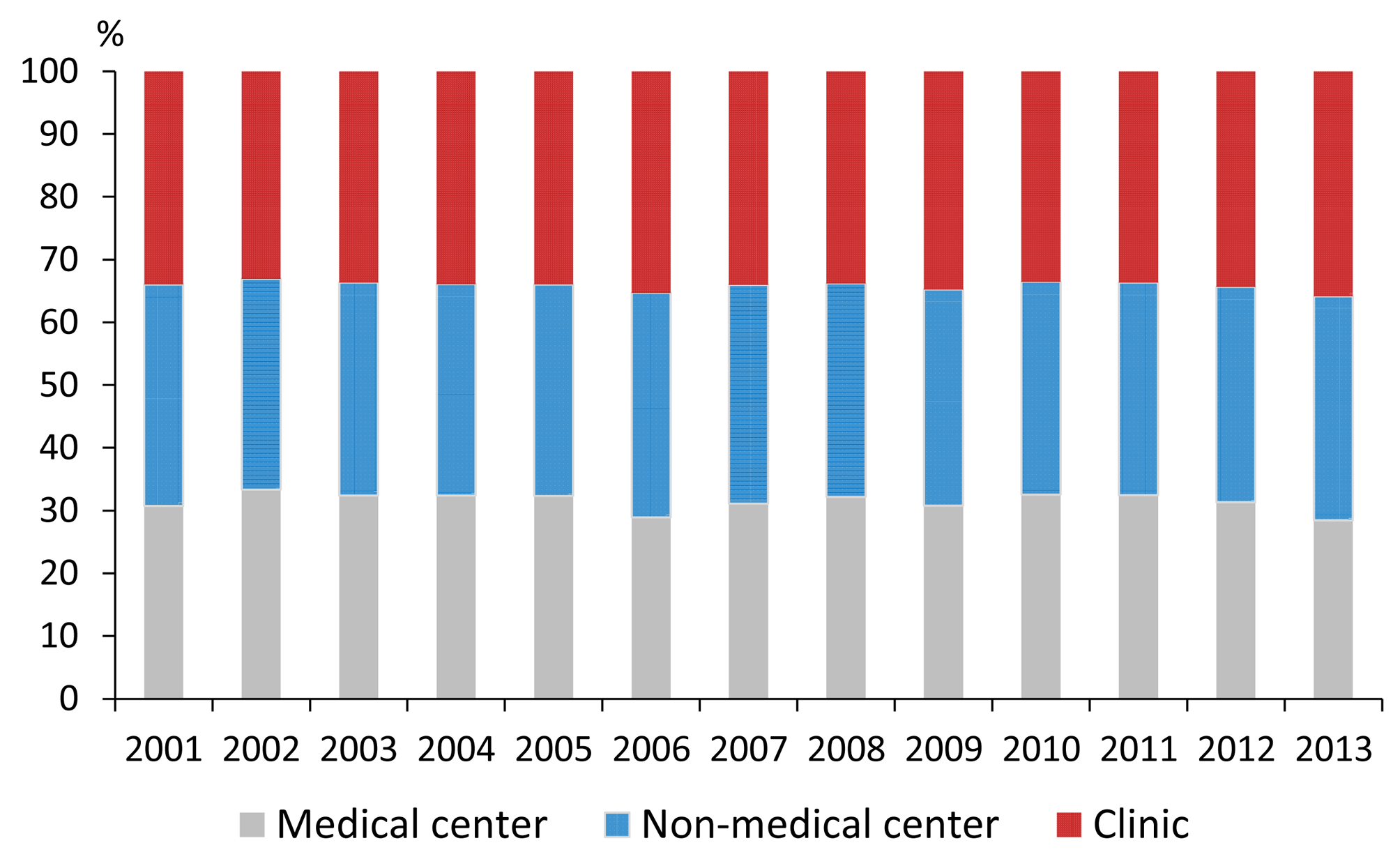

Supplement: Supplementary file 2 — Supplementary Figure S1. [file 41598_2021_177_MOESM2_ESM.tif]
